# Theoretic Study on Dispersion Mechanism of Boron Nitride Nanotubes by Polynucleotides

**DOI:** 10.1038/srep39747

**Published:** 2016-12-22

**Authors:** Lijun Liang, Wei Hu, Zhisen Zhang, Jia-Wei Shen

**Affiliations:** 1 College of Life Information Science and Instrument Engineering, Hangzhou Dianzi University, Hangzhou, People’s Republic of China; 2 Division of Theoretical Chemistry and Biology, School of Biotechnology, KTH Royal Institute of Technology, SE-10691 Stockholm, Sweden; 3 Research Institute for Biomimetic and Soft Matter, Fujian Provincial Key Laboratory of Soft Functional Materials, Department of Physics, Xiamen University, Xiamen, 361005, People’s Republic of China; 4 School of Medicine, Hangzhou Normal University, Hangzhou 310016, People’s Republic of China

## Abstract

Due to the unique electrical and mechanical properties of boron nitride nanotubes (BNNT), BNNT has been a promising material for many potential applications, especially in biomedical field. Understanding the dispersion of BNNT in aqueous solution by biomolecules is essential for its use in biomedical applications. In this study, BNNT wrapped by polynucleotides in aqueous solution was investigated by molecular dynamics (MD) simulations. Our results demonstrated that the BNNT wrapped by polynucleotides could greatly hinder the aggregation of BNNTs and improve the dispersion of BNNTs in aqueous solution. Dispersion of BNNTs with the assistance of polynucleotides is greatly affected by the wrapping manner of polynucleotides on BNNT, which mainly depends on two factors: the type of polynucleotides and the radius of BNNT. The interaction between polynucleotides and BNNT(9, 9) is larger than that between polynucleotides and BNNT(5, 5), which leads to the fact that dispersion of BNNT(9, 9) is better than that of BNNT(5, 5) with the assistance of polynucleotides in aqueous solution. Our study revealed the molecular-level dispersion mechanism of BNNT with the assistance of polynucleotides in aqueous solution. It shades a light on the understanding of dispersion of single wall nanotubes by biomolecules.

Nanotubes, especially carbon nanotubes, due to their unique mechanical and electronical properties, have been extensively investigated in last two decades^1^,^2^,^3^,^4^. Although the chemistry of carbon nanotubes (CNTs) has correspondingly been developed to extend their applicability, there are still many stubborn problems blocking the way toward the real applications of CNTs^5^. In this respect, boron nitride nanotubes (BNNTs) made from BN nanosheet are much more attractive due to their special physicochemical properties^6^,^7^,^8^,^9^,^10^,^11^,^12^,^13^. As a counterpart of a CNT, BNNT possesses notably higher chemical stability and resistance to oxidation^14^,^15^, whereas it exhibits very similar mechanical properties and thermal conductivity^16^. The most interesting thing is that BNNTs are transparent to visible light due to its constant wide band gap (around 5.2–5.8 eV)^17^,^18^,^19^. In addition, Ciofani *et al*. revealed that BNNT has a good cytocompatibility in living cell^20^,^21^. These studies greatly enhanced our understanding on the properties of BNNTs. They show that BNNTs could be used in many potential applications especially nano-biomedical application^22^,^23^,^24^,^25^,^26^,^27^.

To realize these promising application of BNNTs, the aggregation of BNNTs in the aqueous needs to be overcome^28^. In potential applications, dispersion of BNNTs with the assistance of biomolecules is a good choice due to the natural property of biomolecules. Especially, the isolation of BNNTs with the assistance of peptides has been successfully achieved in experiment^29^. Moreover, theoretical studies showed that DNA and polynucleotides has strong interactions with BNNTs^30^,^31^. These studies give a strong hint on the dispersion of BNNT in aqueous with the assistance of DNA or polynucleotides molecules. However, understanding the dispersion mechanism of BNNT by DNA and polynucleotides in aqueous is essential but still unclear. It could give a guideline on the dispersion of single-walled nanotubes by biomolecules. In addition, it could also provide an insight on the fundamental understanding of interaction between biomolecules and nanomaterials.

Besides experiments, molecular dynamics (MD) simulation has been successfully used to investigate the interaction between biomolecules and nanotubes^32^,^33^,^34^,^35^,^36^,^37^,^38^,^39^. MD simulation could provide comprehensive understanding at the atomic level. Recently, Liang *et al*. revealed the insertion process of CNTs into DNA nanotubes by mean of MD simulation^34^. Shen *et al*. found that the chirality of CNTs could greatly affect the interaction between CNTs and polynucleotides^36^. Moreover, density functional theory (DFT) calculations could describe more detailed structures between DNA and CNTs.

Based on this background, MD simulations combined with DFT calculations were used to investigate the interaction between polynucleotides and BNNTs in this study. To investigate the effect of nucleotide type on the dispersion of BNNTs, different types of single strand of polynucleotides including poly(A)_15_, poly(T)_15_, poly(C)_15_ and poly(G)_15_ (abbreviated as A_15_, T_15_, C_15_ and G_15_ in the following of this paper) were used to represent polynucleotides. The details of simulations are listed in the Table 1. Our simulations demonstrate that the BNNT wrapped with polynucleotides could well disperse in aqueous solution and the aggregation of BNNTs could be hindered.

## Computational Methods

### Molecular dynamics simulation

**Figure 1:**
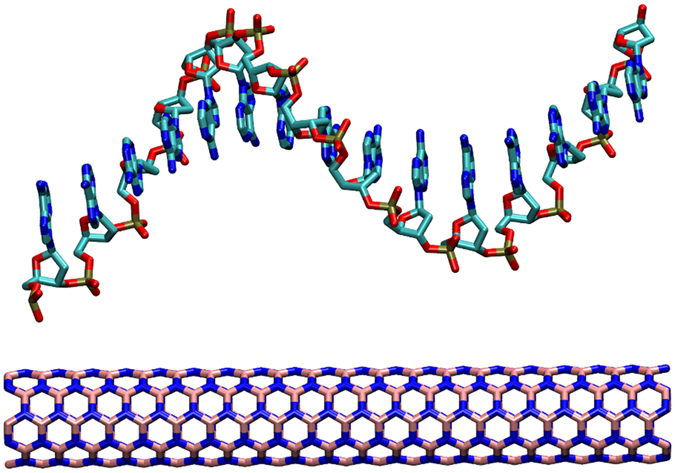
Initial structure for BNNTs (5, 5) with a single strand of poly(A)_15_ from the side view. The polynucleotides molecule and BNNTs are shown by licorice model. The water molecules and ions are not shown for clarity.

The armchair (5, 5) and (9, 9) BNNTs were constructed by visual molecular dynamics (VMD) tool^40^. The diameters of them are 0.69 nm and 1.24 nm with the length of 5.00 nm, respectively. The vertical axis of two BNNTs is along *z* direction. Polynucleotides including A_15_, T_15_, C_15_ and G_15_ were constructed by Hyperchem (Version 7.0, Hypercube, Inc)^41^, and they were equilibrated for 10 ns in MD simulation in vacuum. After that, polynucleotides were immersed into the water box with size of 8.0 × 8.0 × 10 nm^3^, and certain number of Na^+^ ions was added into the water box to neutralize the system. Then 10 ns MD simulation in *NpT* ensemble was performed to equilibrate the system. The structure of polynucleotides at the final state of the equilibration was exacted as the initial structure for investigating the interaction between BNNTs and polynucleotides. The vertical axis of the BNNTs surface was selected to be parallel to the *z*-direction, hence the cross sections of the BNNTs are in the *x*–*y* plane. Then the equilibrated polynucleotides were placed on the top of the BNNTs with the vertical axis of polynucleotides parallel to the vertical axis of BNNTs, and the distance between the center of mass (COM) of polynucleotides and the surface of BNNTs is around 2.0 nm. TIP3P water molecules^42^ were added into the box with system density of 1.002 g/cm^3^. The number of water molecules is slightly different in different system, and the details are displayed in Table 1. At last, Na^+^ ions were added into the solution to neutralize the system. In most cases, the water box is 8.00 × 8.00 × 10.00 nm^3^ in the *x, y* and *z* directions, as shown in Fig. 1.

After the wrapping process of polynucleotides on single BNNTs, the complexes including BNNTs and polynucleotides were used as the initial structure to investigate the effect of polynucleotides on the aggregation process of BNNTs. 9 BNNT-polynucleotides complexes were copied in *x-y* plane. After that, they were immersed into the water box, and around 81, 200 water molecules were added into the box. At last, Na^+^ ions were added into the water box to neutralize the system. The system of 9 BNNT(9, 9)-poly(A)_15_ complexes contains 251, 212 atoms including water molecules, and the total number of atoms in different systems containing MBNNTs is slightly different in different systems, as shown in Table 1.

The polynucleotides, Na^+^ and Cl^−^ ions were modeled by the Charmm27 force field^43^. The force field parameters of boron atoms and nitride atoms in BNNTs were taken from the reference^44^. The charge on N atoms is −0.3*e*, and it is 0.3*e* on B atoms. All atoms including hydrogen atoms were represented explicitly, and the bonds with hydrogen atom were constrained by LINCS algorithm. The non-bonded van der Waals interaction was set by a switching function starting at 1.0 nm and reaching zero at 1.2 nm. The Particle mesh Ewald (PME) summation^45^ was used to calculate the long-ranged electrostatic interactions, with a cutoff distance of 1.2 nm for the separation of the direct and reciprocal space. All simulations were performed by Gromacs-4.6.3 program with the time step of 2 fs. Periodic boundary conditions (PBC) were applied in all MD simulations. After energy minimization and 10 ns equilibration, all MD simulations were carried out in *NpT* ensemble, and the Langevin method was employed to keep the temperature at 298 K and the pressure at 101.3 kPa, respectively.

### Density functional theory calculations

All DFT calculations were performed using PBC model implemented in the Vienna ab initio simulation package (VASP)^46^. The projector augmented wave pseudopotentials were employed to represent the interaction between the core ions and the valence electrons^47^. Meanwhile, the exchange-correlation effects were mainly described by the Perdew−Burke−Ernzerhof generalized-gradient approximation (GGA-PBE)^48^, with a plane-wave basis cutoff of 400 eV. To consider the Van der Walls interaction between nucleotides and BNNT, DFT-D3 method with Becke-Jonson damping was used^49^. The structures of different nucleotides adsorbed on BNNT with different radius were first optimized. The interaction energy between nucleotides and BNNT was defined as:


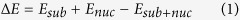


where *E*_*sup*_ represents the energy of BNNT, *E*_*nuc*_ and *E*_*sub*+*nuc*_ represents the energy of the nucleotides and the complexed system (eg. nucleotide + BNNT), respectively. To avoid the contribution of the preparation energy, *E*_*sub*_ and *E*_*nuc*_ were calculated from the restricted geometries which were extracted from the corresponding structures of the optimized complexed systems^50^.

## Results and Discussion

### Wrapping of polynucleotides around BNNTs

**Figure 2:**
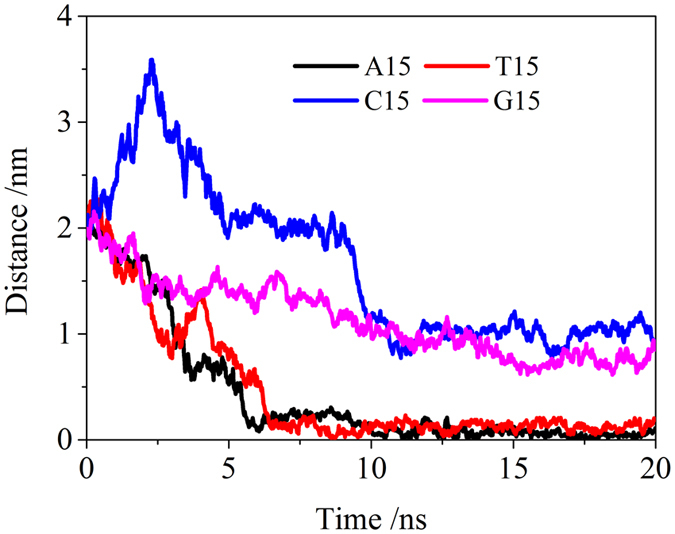
The distance between the center of mass of polynucleotides (A_15_, T_15_, C_15_, and G_15_) and the surface of single BNNT(9, 9) in *x-y* plane.

**Figure 3:**
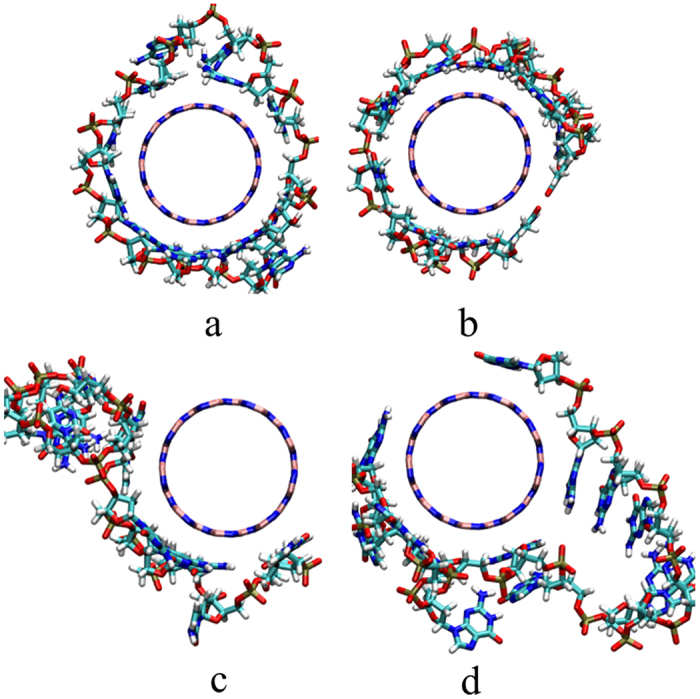
The wrapping conformation of polynucleotides on BNNT(9, 9) at the end of simulation. (**a**) A_15_; (**b**) T_15_; (**c**) C_15_ and (**d**) G_15_.

**Figure 4:**
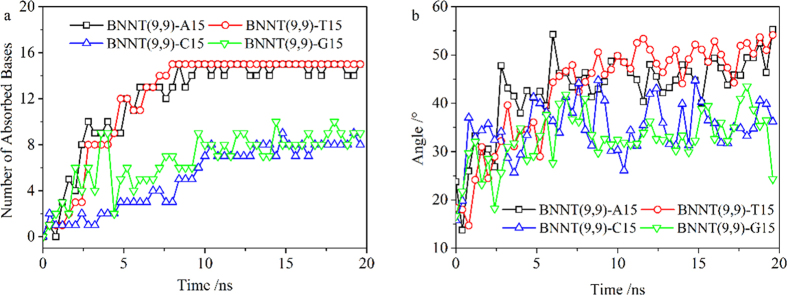
(**a**) The number of adsorbed bases of polynucleotides (including A_15_, T_15_, C_15_ and G_15_) on BNNT(9, 9) as a function of simulation time. (**b**) The averaged angle between bases of polynucleotides and the *x-y* plane of BNNT(9, 9) with respect to simulation time.

The distance between center of mass of polynucleotides and the center of mass of BNNT (9, 9) was measured and displayed in Fig. 2. Herein, to investigate the packing manner of polynucleotides on BNNT (9, 9), only the *x* and *y* components in the center of mass were calculated. As shown in Fig. 2, the distance between COM of all different polynucleotides to the surface BNNT (9, 9) decreases in the simulation. The distance between G_15_ and BNNT(9, 9) is about 0.91 nm, and it is around 1.03 nm between C_15_ and BNNT(9, 9). Especially, the distances from A_15_ and E_15_ to the surface of BNNT(9, 9) are both less than 0.2 nm. It shows that all polynucleotides could adsorb on BNNT (9, 9) surface. The packing manner of polynucleotides including A_15_, T_15_, C_15_ and G_15_ on the BNNT (9, 9) were observed in the simulation. As seen in Fig. 3, the wrapping conformation of polynucleotides on the BNNT (9, 9) was extracted after 20 ns simulation. A_15_ and T_15_ could tightly surrounded the BNNT (9, 9), as seen in Fig. 3a and b. As shown in Fig. 3c and d, C_15_ and G_15_ can also adsorb on BNNT (9, 9) but only part of BNNT (9, 9) was tightly surrounded by C_15_ or G_15_. It implies that the wrapping manner of different polynucleotides on BNNT (9, 9) is different. The wrapping of A_15_ and T_15_ on BNNT(9, 9) is much better than C_15_ and G_15_. It was confirmed by the adsorption number of bases and angle of polynucleotides on BNNT(9, 9) from the last 5 ns simulation. Herein, the adsorption base was defined as: distance between COM of one base and the surface of BNNT(9, 9) is less than 6.0 Å, as described in our previous work^51^. The angle is calculated between the base plane of polynucleotides and *x-y* plane of BNNT(9, 9). As seen in Fig. 4, the number of adsorbed bases of A_15_ and T_15_ on BNNT(9, 9) is more than that of C_15_ and G_15_ on BNNT(9, 9). The angle between bases of A_15_ and T_15_ and BNNT(9, 9) is larger than that of C15 and G15. It implicated that the bases of A_15_ and T_15_ is more favorable to adopt the orientation that parallel to the BNNT (9, 9), comparing to the bases of C_15_ and G_15_. It confirmed that the bases of A_15_ and T_15_ tend to stack on the surface of BNNT (9, 9). In addition, as seen in Fig. 3, all the bases are closely adsorbed on the surface of BNNT(9, 9), and the phosphate groups are arranged to be far away from the surface of BNNT(9, 9). The adsorption behaviors of polynucleotides on BNNT(9, 9) is quite similar to that of polynucleotides on neutral carbon nanotubes (CNTs)^35^.

**Figure 5:**
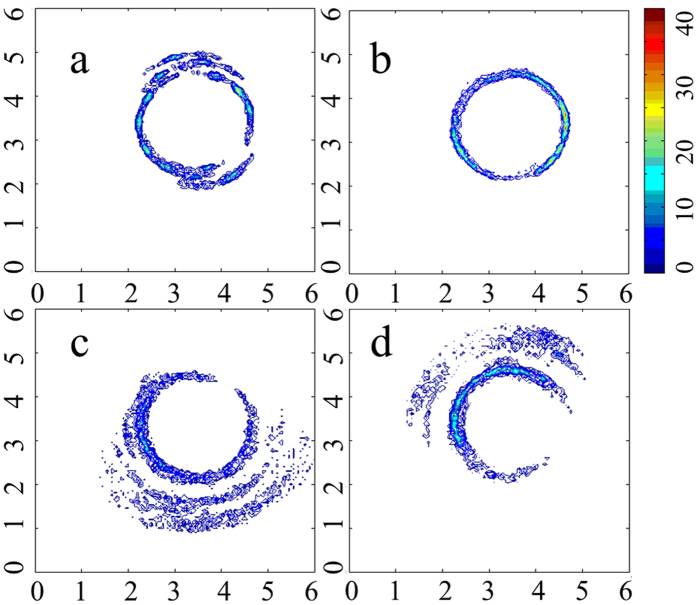
Density of phosphorus atoms of polynucleotides around BNNT (9, 9) in *x-y* plane. (**a**) A_15_; (**b**) T_15_; (**c**) C_15_; (**d**) G_15_.

To understand the wrapping manner of polynucleotides on BNNT(9, 9) more deeply, the density of phosphorus atoms around BNNT (9, 9) in *x-y* plane from the last 5 ns simulation was measured. The density of phosphorus atoms of polynucleotides in *x-y* plane is along the surface of BNNT(9, 9). Especially, the shape of density distribution of phosphorus atoms is quite similar to the shape of BNNT(9, 9) in *x-y* plane in systems with A_15_ (Fig. 5a) and T_15_ (Fig. 5b). There is only one layer of phosphorus atoms distributed on the surface of BNNT(9, 9) in the systems with A_15_ or T_15_. It implicated that BNNT(9, 9) is tightly surrounded by A_15_ or T_15_, and all the bases of A_15_ or T_15_ interacted strongly with BNNT(9, 9). For the system with C_15_ (Fig. 5c) or G_15_ (Fig. 5d), the BNNT(9, 9) was also tightly surrounded by part of polynucleotides but not the whole polynucleotides. Therefore, the interaction between the rest polynucleotides and BNNT(9, 9) is relatively weak due to the unpacked interaction manner.

**Figure 6:**
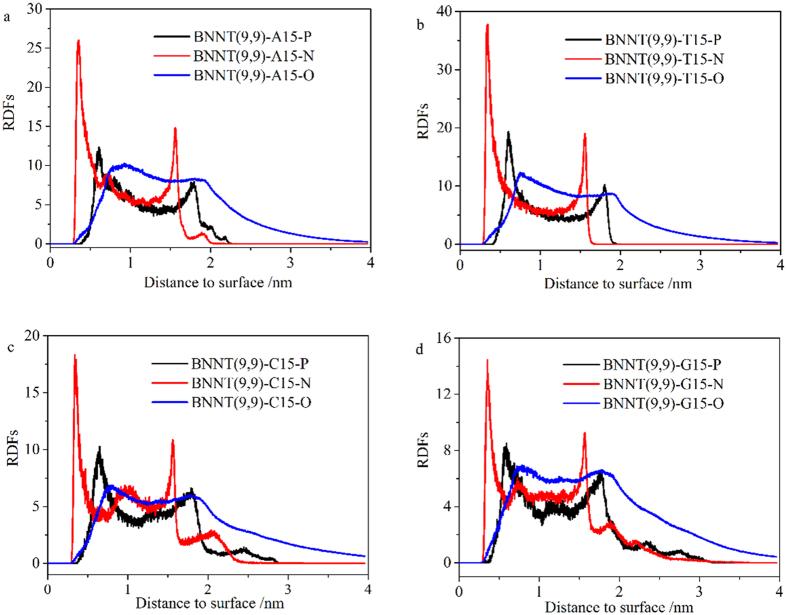
Density of phosphorus atoms and oxygen atoms in phosphate group and nitride atoms in base on the x-y plane of BNNT(9, 9) in different systems: (**a**) A_15_; (**b**) T_15_; (**c**) C_15_ and (**d**) G_15_.

**Figure 7:**
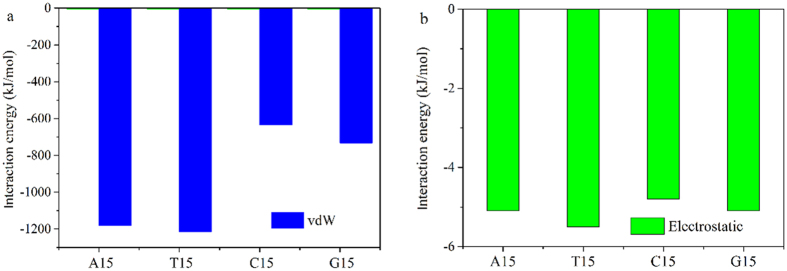
The interaction energy between polynucleotides and BNNT(9, 9): (**a**) vdW interaction energy (blue) and (**b**) electrostatic interaction energy (green).

To better understand the dispersion mechanism of BNNT by polynucleotides, the density of phosphorus and oxygen atoms in phosphate group, nitride atoms in base to the surface of BNNT(9, 9) was measured, as shown in Fig. 6. The first peak of nitride atoms is about 0.35 nm to the surface of BNNT(9, 9), and it is about 0.60 nm of phosphorus atoms to the surface of BNNT(9, 9) in all systems. It reveals that the base of polynucleotides but not phosphate groups is adsorbed on the surface of BNNT(9, 9) in all systems. It implicated that pi-pi interaction between bases and BNNT(9, 9) is important for the adsorption of polynucleotides on BNNT(9, 9), and the phosphate groups were relatively far from the surface of BNNT(9, 9). The adsorption behavior of polynucleotides on BNNT(9, 9) is similar to the adsorption behavior of polynucleotides on CNT (10, 10)^48^. Similar to adsorption of polynucleotides on neutral CNT (10, 10), the adsorption of polynucleotides also relies on the base of polynucleotides on positively charged boron atoms and negatively charged nitride atoms. To investigate the importance of van der Waals (vdW) interaction on adsorption of polynucleotides on BNNT(9, 9), the interaction between BNNT(9, 9) and different polynucleotides from the last 5 ns simulation were measured, as shown in Fig. 7. Herein, the total interaction between polynucleotides and BNNT(9, 9) was divided into two parts: vdW interaction and electrostatic (Ele) interaction. The percentages of vdW interaction are more than 90% in all systems. Moreover, the change of both vdW and electrostatic interaction during last 5 ns in MD simulation in these systems were displayed in Figure S1. These results implicated that the vdW interaction is most important interaction between polynucleotides and BNNT(9, 9).

### Dispersion of multiple BNNTs

**Figure 8:**
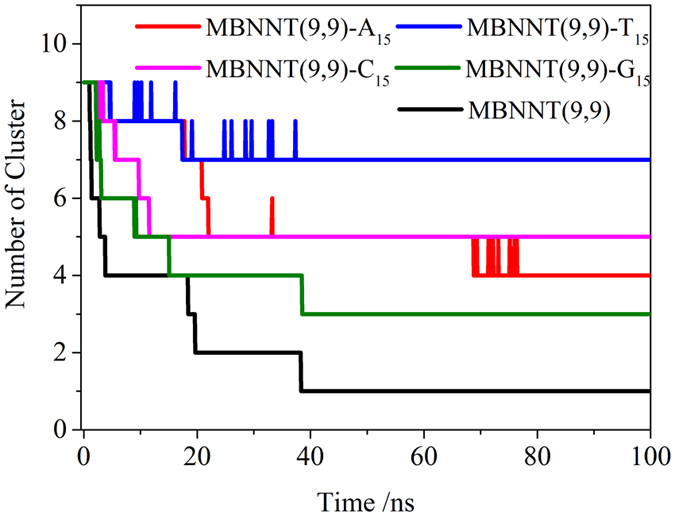
The change of number of cluster as a function of simulation time in the system of multiple BNNT(9, 9) in the solution of polynucleotides.

**Figure 9:**
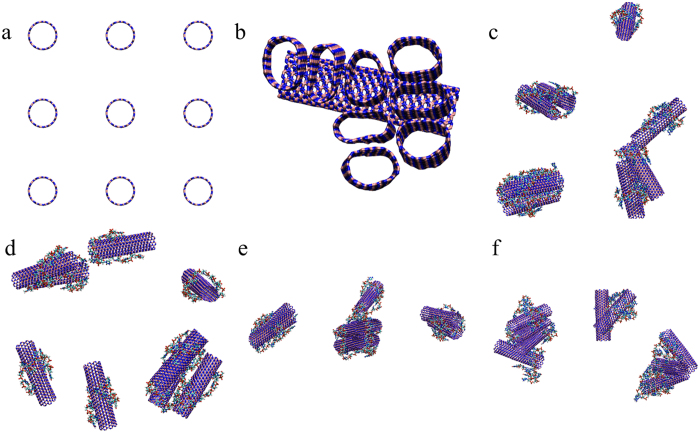
The snapshots of multiple BNNT(9, 9) in the simulation. The BNNTs and polynucleotides are represented by licorice model. (**a**) Initial structure of 9 BNNT(9, 9) in all systems; the last structure of 9 BNNT(9, 9) in the system of (**b**) MBNNT(9, 9), (**c**) MBNNT(9, 9)-A_15_; (**d**) MBNNT(9, 9)-T_15_; (**e**) MBNNT(9, 9)-C_15_; (**f**) MBNNT(9, 9)-G_15_ in the simulation.

To investigate the effect of polynucleotides on the dispersion of BNNT(9, 9) in aqueous solution, the simulations (details in Table 1) including 9 BNNT(9, 9) with or without polynucleotides were performed. The number of cluster in different systems with respect to the simulation time is measured, as shown in Fig. 8. The multiple BNNT(9, 9) without polynucleotides could aggregated into one cluster, which was confirmed by the experiment^25^. It is the reason that the isolation of single wall BNNT(9, 9) in aqueous solution is very difficult. With the assistance of polynucleotides, the number of cluster is more than one in all other systems. As seen in Fig. 9, the conformations of different systems at the end of 100 ns simulation were displayed. It assembled into 3 aggregates under the assistance of G_15_, 4 aggregates under the assistance of A_15_, 5 aggregates under the assistance of C_15_, and 7 aggregates under the assistance of T_15_, respectively. Especially in the system of MBNNT(9, 9)-T_15_, 9 BNNT(9, 9) aggregated into 7 clusters. It strongly implicates that BNNT(9, 9) could disperse in aqueous solution under the assistance of polynucleotides, and the dispersion of BNNT(9, 9) could be affected by the wrapping manner of polynucleotides. As shown in Figure S2, it could be found that the base of polynucleotides is close to MBNNT(9, 9) surface, and phosphate group of polynucleotides prefer to interact with water molecules. It mainly due to the fact that the negative charge of phosphate group could strongly interact with water molecules, and thus it could stabilize the dispersion of BNNT(9, 9) in aqueous. Especially, the number of cluster of MBNNT(9, 9) with T_15_ is 7. It indicate that BNNT(9, 9) with T_15_ could well disperse in aqueous solution. From the data of Fig. 5, the whole BNNT(9, 9) was circled by T_15_ through aromatic bases, and all phosphate groups of T_15_ is annularly distributed outside and interact with water molecules.

### Effect of radius of BNNT on dispersion

To investigate the dispersion of BNNT by polynucleotides and the effect of radius of BNNT on dispersion, the MD simulation of systems with small radius of BNNT (BNNT(5, 5)) and polynucleotides (A_15_, T_15_, C_15_ and G_15_) were performed. As seen in Figure S3, the BNNT(5, 5) could be wrapped by all polynucleotides (A_15_, T_15_, C_15_ and G_15_). However, comparing with the wrapping of polynucleotides on BNNT(9, 9), not the whole circle of BNNT(5, 5) was surrounded by polynucleotides. It shows that the wrapping of polynucleotides on BNNT(9, 9) is much stronger than that of BNNT(5, 5). It could also be confirmed from the density distribution of phosphorus atoms of polynucleotides around BNNT (5, 5) in *x-y* plane, as seen in Figure S4. In addition, the aggregation process of 9 BNNT(5, 5) with polynucleotides in aqueous were observed. In Figure S5, the number of cluster of BNNT(5, 5) during the simulation was measured. The number of cluster of BNNT(5, 5) is 2 in both systems with A_15_ and T_15_. It shows that 9 BNNT(5.5) aggregates in aqueous solution even with the assistance of polynucleotides. These results implicated that the strength of the interaction between polynucleotides and BNNT could certainly affect by the curvature of BNNT, and thus the dispersion of BNNT by polynucleotides is highly relate to the radius of BNNT.

The radius of BNNT(5, 5) is 0.35 nm, which is smaller than that of polynucleotides (~0.5 nm). The radius of BNNT(9, 9) is 0.62 nm, which is larger than that of polynucleotides. Therefore, the wrapping of polynucleotides on whole BNNT(5, 5) is more difficult than that on BNNT(9, 9). To understand the wrapping of nucleotides on BNNT from the view of adsorption energy, the interaction between single nucleotide (A and T) and BNNT including BNNT(5, 5) and BNNT(9, 9) was calculated by density functional theory (DFT), as shown in Table 2. The interaction between A and BNNT(5, 5) is around 64.02 kJ/mol, and it is 74.06 kJ/mol between A and BNNT(9, 9). The interaction between T and BNNT(5, 5) is around 67.78 kJ/mol, and it is 73.22 kJ/mol between A and BNNT(9, 9). It reveals that the interaction between nucleotides and BNNT(9, 9) is larger than that between nucleotides and BNNT(5, 5). It implicated that the radius of BNNT could affect the interaction between nucleotide and BNNT, and sequentially affects the wrapping of nucleotides on BNNT.

## Conclusions

In summary, the molecular-level dispersion mechanism of BNNT in aqueous solution by polynucleotides was uncovered by molecular dynamics simulation. Our results showed that BNNT could be wrapped by polynucleotides in aqueous solution with the interaction between bases of polynucleotides and surface of BNNT. Polynucleotides have hydrophobic groups (base) and the hydrophilic groups (phosphate groups). The base of polynucleotides tends to closely interact with the surface of BNNT, and the phosphate atoms could interact with water molecules. Therefore, the phosphate groups of polynucleotides could assistant BNNT dispersing in aqueous and hinder the aggregation of BNNT. Meanwhile, the dispersion of BNNT in aqueous solution is greatly affected by the type of polynucleotides. Moreover, the radius of BNNT could also affect the dispersion of BNNT in aqueous solution by changing the interaction strength between nucleotides and BNNT. This work could help to understand the dispersion mechanism of BNNT in aqueous solution by the assistance of polynucleotides, and it may shade a light on the research of separation of single wall nanotubes by biomolecules.

## Additional Information

**How to cite this article**: Liang, L. *et al*. Theoretic Study on Dispersion Mechanism of Boron Nitride Nanotubes by Polynucleotides. *Sci. Rep.* **6**, 39747; doi: 10.1038/srep39747 (2016).

**Publisher's note:** Springer Nature remains neutral with regard to jurisdictional claims in published maps and institutional affiliations.

## Supplementary Material

Supplementary Information

## Figures and Tables

**Table 1:** The details of performed systems in this study.

System	BNNT	Radius of BNNT (nm)	polynucleotides	Atoms	Simulation time (ns)
MD simulations					
BNNT(9, 9)-A_15_	Single BNNT(9, 9)	0.620	A_15_	63, 559	20
BNNT(9, 9)-T_15_	Single BNNT(9, 9)	0.620	T_15_	63, 544	20
BNNT(9, 9)-C_15_	Single BNNT(9, 9)	0.620	C_15_	63, 559	20
BNNT(9, 9)-G_15_	Single BNNT(9, 9)	0.620	G_15_	63, 592	20
MBNNT(9, 9)	9 BNNT(9, 9)	0.620	/	194, 084	100
MBNNT(9, 9)-A_15_	9 BNNT(9, 9)	0.620	A_15_	252, 212	100
MBNNT(9, 9)-T_15_	9 BNNT(9, 9)	0.620	T_15_	252, 917	100
MBNNT(9, 9)-C_15_	9 BNNT(9, 9)	0.620	C_15_	253, 049	100
MBNNT(9, 9)-G_15_	9 BNNT(9, 9)	0.620	G_15_	252, 674	100
BNNT(5, 5)-A_15_	Single BNNT(5, 5)	0.345	A_15_	35, 567	20
BNNT(5, 5)-T_15_	Single BNNT(5, 5)	0.345	T_15_	35, 585	20
BNNT(5, 5)-C_15_	Single BNNT(5, 5)	0.345	C_15_	35, 546	20
BNNT(5, 5)-G_15_	Single BNNT(5, 5)	0.345	G_15_	35, 564	20
MBNNT(5, 5)-A_15_	9 BNNT(5, 5)	0.345	A_15_	213, 745	100
MBNNT(5, 5)-T_15_	9 BNNT(5, 5)	0.345	T_15_	214, 871	100
**DFT calculations**					
BNNT(9, 9)-A		Single BNNT(9, 9)		A	
BNNT(9, 9)-T		Single BNNT(9, 9)		T	
BNNT(5, 5)-A		Single BNNT(5, 5)		A	
BNNT(5, 5)-T		Single BNNT(5, 5)		T	

**Table 2:** The interaction between nucleotide and BNNTs (kJ/mol).

	A	T
BNNT(5, 5)	64.02	67.78
BNNT(9, 9)	74.06	73.22
