# Supplementary material for: Theoretic Study on Dispersion Mechanism of Boron Nitride Nanotubes by Polynucleotides
Source: Sci Rep. 2016 Dec 22;6:39747. doi: 10.1038/srep39747 (PMC5177943; doi:10.1038/srep39747)
Supplement: Supplementary Information [file srep39747-s1.docx]

**Supporting Information**

**Theoretic Study on Dispersion Mechanism of Boron Nitride Nanotubes by Polynucleotides**

Lijun Liang^1,#^, Wei Hu^2,#^, Zhisen Zhang^3,*^, Jia-Wei Shen^4,*^

^1^College of Life Information Science and Instrument Engineering, Hangzhou Dianzi University, Hangzhou, People's Republic of China

^2^Division of Theoretical Chemistry and Biology, School of Biotechnology, KTH Royal Institute of Technology, SE-10691 Stockholm, Sweden

^3^ Research Institute for Biomimetic and Soft Matter, Fujian Provincial Key Laboratory of Soft Functional Materials, Department of Physics, Xiamen University, Xiamen, 361005, People’ s Republic of China

^4^School of Medicine, Hangzhou Normal University, Hangzhou 310016, People’s Republic of China

^#^ These authors contributed equally to this work.

* Corresponding authors.

Tel: +86-87952424; Fax: +86-87952424 (L. Liang)

E-mail addresses:

shen.jiawei@hotmail.com (J.W. Shen)

[zhangzs@xmu.edu.cn](mailto:zhangzs@xmu.edu.cn) (Z. Zhang)

Figure S1. The interaction energy between polynucleotides and BNNT(9, 9) as a function of simulation time in the last 5ns: (A) vdW interaction energy; (B) electrostatic interaction energy.


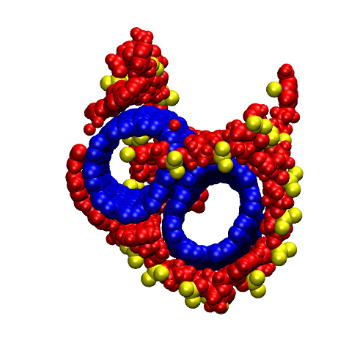


Figure S2. The snapshot of the wrapping of poly(A)_15_ and BNNTs. The BNNT was shown by blue vdW model, the phosphate group of polynucleotides was shown in yellow vdW model, and the base of polynucleotides was shown in red vdW model.


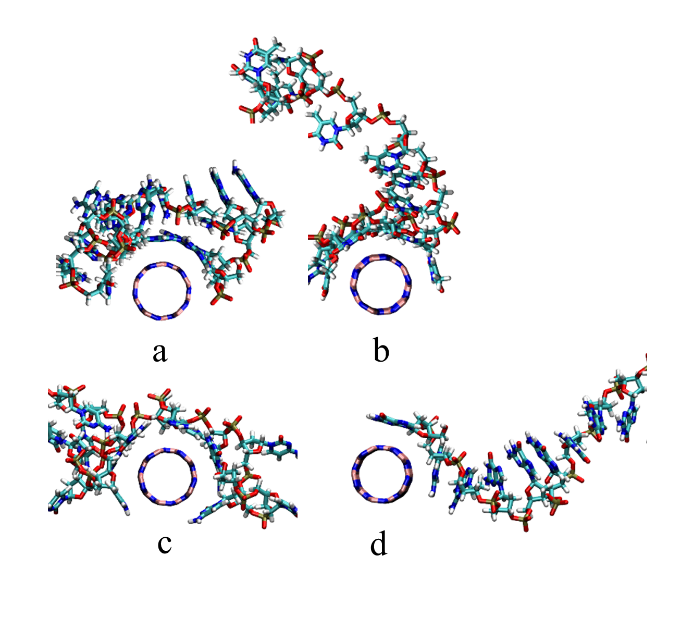


Figure S3. The wrapping conformation of polynucleotides on BNNT(5, 5) at the end of MD simulation. (a) A_15_; (b) T_15_; (c) C_15_ and (d) G_15_.


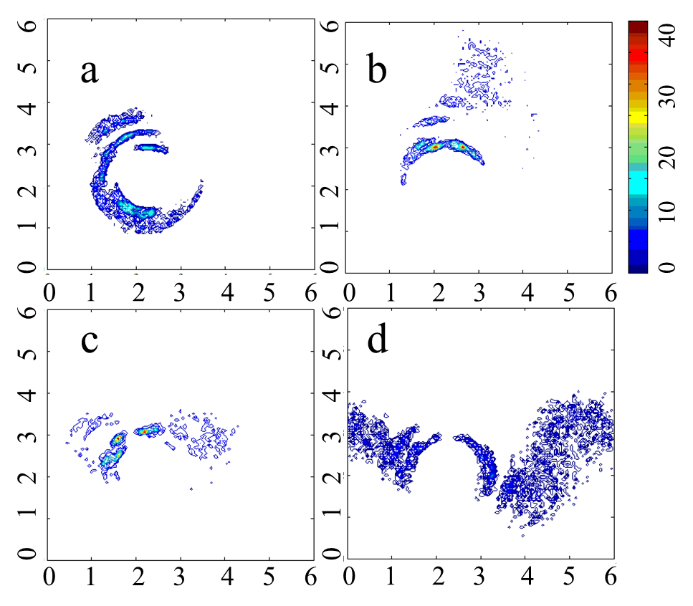


Figure S4. Density of phosphorus atoms of polynucleotides around BNNT (5, 5) in *x-y* plane. (a) A_15_; (b) T_15_; (c) C_15_; (d) G_15_.

Figure S5. The change of number of cluster as a function of simulation time in systems of MBNNT(5,5)-polynucleotides.
